# Does Health-Based Prospective Risk Adjustment Adequately Compensate for Individuals Diagnosed With a New Chronic Disease?

**DOI:** 10.1177/10775587251378167

**Published:** 2025-11-14

**Authors:** Michel Oskam, Richard C. van Kleef, René C. J. A. van Vliet

**Affiliations:** 1 Erasmus School of Health Policy & Management, Erasmus Centre for Health Economics Rotterdam, Erasmus University Rotterdam, Rotterdam, The Netherlands

**Keywords:** health insurance, risk adjustment, risk selection, risk equalization

## Abstract

Many regulated health insurance markets use prospective risk adjustment (RA) to mitigate risk selection incentives for insurers. However, prospective RA might underpay insurers for people diagnosed with a new chronic disease. By tracking spending and RA payments over the period *t*−2 to *t*+2 for individuals diagnosed with a new chronic disease in year *t*, we find a substantial payment gap in year *t* and, to a lesser extent, in prior and/or subsequent years. The extent to which these gaps stimulate selection incentives for insurers depends on the possibilities for insurers to distort consumers’ choice of insurance products. Possibilities which—in turn—depend on whether and when consumers respond to the onset of the chronic disease when choosing an insurance product. By analyzing “insurer switching” in the period *t*−2 to *t*+2 we find that—on average—people first diagnosed with a chronic disease are more likely to switch insurer than others.

## Introduction

Around the world many health insurance markets are based on principles of regulated competition (McGuire & Van Kleef, 2018). Examples include the basic health insurance schemes in the Netherlands, Germany and Switzerland, and the Marketplaces in the United States under the Affordable Care Act, as well as Medicare Advantage. Economic theory suggests that a balanced combination of competition and regulation can enhance efficiency and fairness of health insurance markets (Enthoven, 1978, 1988). Typical aspects of regulation include standardization of coverage, open enrollment requirements and premium rate restrictions. However, while such regulations enhance the accessibility and affordability of health insurance for individuals with a high risk of medical spending, they can exacerbate selection problems (Van de Ven & Ellis, 2000). For example, mandating insurers to charge identical premiums to individuals for an insurance contract, irrespective of age, health status and prior health spending, creates predictable profits and losses which confront insurers with incentives for risk selection (Glazer & McGuire, 2000; Newhouse, 1996; Pauly & Langwell, 1983; Van de Ven et al., 2017). Possible selection actions range from subtle marketing strategies to distortions of benefits, cost-sharing schedules and provider networks. For example, health insurers might choose not to contract high-quality providers that treat disproportionate shares of unprofitable risk types (Bauhoff et al., 2017; Decoralis & Guglielmo, 2017; Shepard, 2022; Stolper et al., 2022; Van de Ven et al., 2015). These actions could seriously undermine the functioning of health (insurance) systems through skimping on quality and access to health care (Einav & Finkelstein, 2011; Van Kleef et al., 2024).

To mitigate risk selection by insurers, regulators have implemented schemes of prospective risk adjustment (RA) to compensate insurers, based on estimations of predictable variation in health spending between individuals (Ellis et al., 2018). Over the past decades, prospective RA models have evolved from simple demographic models to sophisticated health-based models with risk adjusters based on diagnostic information, socioeconomic variables and/or prior health care utilization/spending. The uptake of health information has significantly improved the predictive strength of RA models, leading to more accurate compensation of insurers and reducing incentives for risk selection (Layton et al., 2018; Van Kleef et al., 2018).

Despite the improvements, it is questionable whether prospective RA models adequately compensate insurers for a specific group of enrollees: those who develop a (chronic) disease. The reason is threefold. First, individuals who develop a disease are not immediately diagnosed. Initial examinations and treatments may precede the eventual diagnosis and lead to elevations in health care utilization and spending. Therefore, for as long as the diagnosis has not been registered, the RA model might not adequately nor timely acknowledge the rise in health spending through higher compensations. Second, in prospective RA health spending of individuals is predicted using health care utilization in the preceding year, creating a time lag in the compensation of insurers (e.g., diagnoses from year *t*−1 are used to predict health spending in year *t*). As a result, a diagnosis of a chronic disease made in year *t*—and its related inflated health spending—will be recognized by the RA model in year *t*+1 at the earliest, leaving a potential financial loss to insurers in the years prior. Third, in the first years after diagnosis, individuals who develop a chronic disease in general require more examinations and treatments than those for whom the disease has stabilized over time. So, within the group of individuals with a chronic disease, those recently diagnosed might be, on average, costlier to insurers than the rest of the group and yield inadequate compensation.

In this paper, we track the performance of prospective RA for the group of individuals diagnosed with a (new) chronic disease in year *t*. More specifically, the first goal of this study is to calculate the mean financial result after prospective RA in years *t*−2, *t*−1, *t*, *t*+1, and *t*+2 for individuals who are diagnosed with a new chronic disease in year *t*.

The extent to which a negative or positive financial result for insurers regarding consumers who are diagnosed with a new chronic disease is problematic depends on whether insurers are able to steer the behavior of those consumers in the choice of insurance product. In turn, the potential to distort the decision-making depends on whether, when, and how the individual in question responds to the change in health status. So, the more consumers let the change in health status reflect in their choice of insurance plan ***before*** a year in which they are unprofitable to insurers, the stronger will be the incentive for insurers to manipulate their enrollment. Therefore, to expand on the first goal of this study, we track the consumer response of the group of individuals diagnosed with a new chronic disease in year *t*. More specifically, the second goal is to track insurer-switching behavior^1^ in year *t*−2 to year *t*+2 of individuals diagnosed with a new chronic disease in year *t*.

Our study is based on the Dutch basic health insurance. This health insurance is mandatory for every person who lives or works in the Netherlands (over 18 million people in 2025). The benefit package is standardized and includes primary care, inpatient and outpatient hospital care, and pharmaceutical care, among other services. Each year consumers can switch insurance plan. Although the benefit package is standardized, plans can differ in terms of provider network and coverage for out-of-network spending. Through selective contracting, preferred provider contracts and supplementary insurance plans, competing insurers can differentiate themselves to attract enrollment. Plans with more generous networks are typically more expensive than restricted plans but, through the open enrollment policy for every new calendar year, insurers are mandated to accept any applicant for insurance.

The starting point of our empirical analysis is the Dutch RA model, a sophisticated RA model that includes a broad spectrum of risk adjusters based on age, sex, health and socioeconomic variables. The health indicators used in the Dutch model are of a prospective nature. Although the empirical findings are specifically relevant for the Netherlands, our conceptual points are potentially relevant for any country that relies on a prospective RA formula to mitigate selection problems in regulated health insurance markets.

This paper is organized as follows. The “New contribution” section explains how this study provides new insights in relation to existing literature. In the conceptual framework we explain how gaps between spending and RA payments for individuals diagnosed with a new chronic disease can be problematic in light of risk selection. Next, we describe the Dutch context, and the data and methods used for our analysis. We then present the main findings. Finally, we discuss the implications of our findings for the design of health insurance markets.

## New Contribution

Over the past decades, many papers have been published on the design and evaluation of RA in health insurance markets. A typical method to evaluate the extent to which an RA model reduces selection incentives is to simulate the financial gaps after RA for selective groups of (un)healthy enrollees (see, for instance, McGuire, Schillo, & Van Kleef, 2021; Van Kleef et al., 2019; Zink & Rose, 2021). Ideally, these evaluations focus on groups that are vulnerable to selection actions by insurers. For example, in their evaluations of American RA models, Montz et al. (2016) use mental health disorder diagnoses and Geruso et al. (2019) use prescribed drugs to identify subgroups that are potential selection targets. Other researchers used diagnostic information from electronic patient records (e.g., Oskam et al., 2023; Van Kleef et al., 2020) or health survey information to identify individuals with specific chronic diseases (e.g., McWilliams et al., 2012; Withagen-Koster et al., 2023).^2^ Although the subdivisions of the population in these studies provide a meaningful basis for evaluating selection incentives, such subgroups defined by the presence of a chronic disease can still be relatively heterogeneous. One dimension of heterogeneity is the time passed since a diagnosis was first registered. This study evaluates the outcomes of a sophisticated RA model over a 5-year period for individuals who are diagnosed with a new chronic disease, setting it apart from prior studies.

In addition to measuring the RA performance, this study examines the insurer-switching behavior of individuals who are diagnosed with a new chronic disease. Over the past decades, several papers have been published on health plan switching and its relation to health status (Boonen et al., 2016; Cunningham & Kohn, 2000; Ellis et al., 2017; Nuscheler & Knaus, 2005; van Vliet, 2006). Individuals who (anticipate to) incur a health shock are likely to reconsider their insurance plan (Croes et al., 2025; Cutler & Zeckhauser, 2000; Einav et al., 2013; Ellis, 1989; Robinson et al., 1993; Van de Ven & Van Praag, 1981). For instance, in a study on Chinese health insurance, Xu et al. (2016) have found increased uptake of insurance for individuals who had recently fallen ill or developed a chronic illness. Similarly, a positive association was found between lagged utilization of services by a general practitioner (GP) or a specialist and the purchase of supplementary private health insurance in Ireland (Bolhaar et al., 2012). Lange et al. (2017) have found evidence of a higher propensity to renew supplementary hospital insurance in Germany for individuals with a below-average health status, and Bajari et al. (2014) have found that individuals in more generous insurance plans face a greater risk of a health shock than others. It should be noted that these findings are dependent on the context and can vary from system to system.^3^

These differences in the latent health status of enrollees between plans provide an indication of consumer behavior in anticipation of, or in response to, a change in health status. Our study adds to this literature by examining the switching behavior of individuals who are diagnosed with a new chronic disease. We go beyond the existing literature by combining the consumer response in terms of insurer switching of this group with multi-year patterns in spending and RA payments. To the best of our knowledge, this focus has not been applied in earlier research. In the conceptual framework, we elucidate why the combined approach is crucial for evaluating the functioning of the health insurance market.

## Conceptual Framework

**Figure 1.**
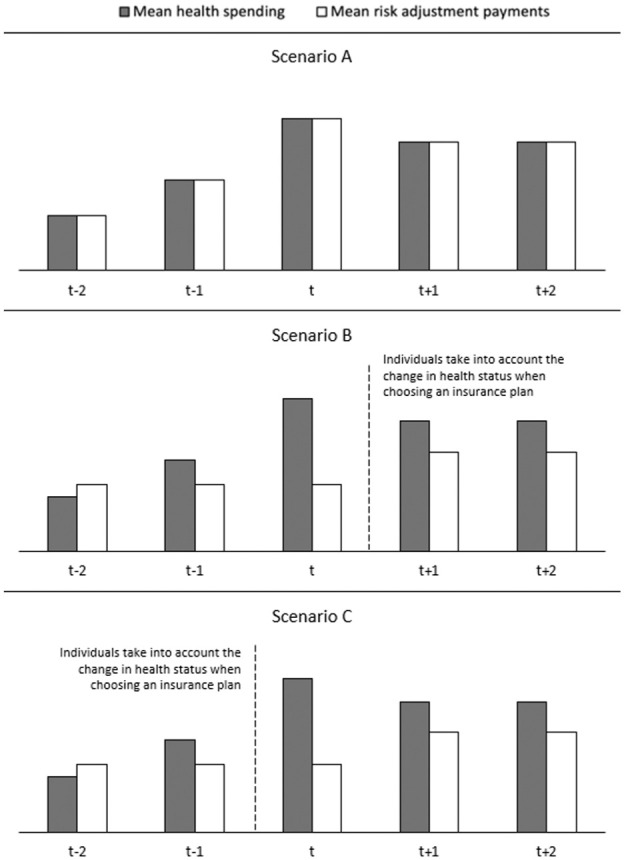
Three Hypothetical Scenarios of Mean Health Spending, Risk Adjustment Payments, and Switching Patterns Over a 5-Year Period, for Individuals Who Are Diagnosed With a New Chronic Disease in Year t. Note: For simplicity, premiums are ignored in the figure. As a result, all financial compensation received by insurers is through RA payments.

This section elaborates on the two main factors that determine incentives for insurers to select against individuals diagnosed with a new chronic disease: (1) the outcomes of the RA model and (2) the response of these individuals to their change in health status. This can be illustrated with Figure 1, which presents a simplified hypothetical scenario of the evolution of health spending over 5 consecutive years for a group of people who are newly diagnosed with a chronic disease in year *t*. In the three panels of the figure, the pattern in mean health spending is identical: before the diagnosis is first registered (year *t*) mean spending starts to rise (e.g., due to the first signals of the upcoming disease); in year *t* mean spending peaks (e.g., due to extensive health checks and treatments); after year *t* mean spending stabilizes, though at a higher level than before the onset of the disease (e.g., due to structural treatment).

A vital first question on selection incentives for insurers is how RA payments develop over the considered 5-year period. For illustrative reasons, Figure 1 distinguishes three scenarios. In Scenario A, the mean RA payment for the group of interest perfectly follows the mean spending. In this scenario, there are no (predictable) profits and losses for this group, implying the absence of selection problems regarding this group.

However, it is unlikely that RA payments perfectly match spending for the group of interest in reality. Scenarios B and C demonstrate notable gaps between health spending by consumers and the compensation received by insurers. As a result of the belated initiation of compensation through prospective RA, a significant payment gap is expected in year *t*. For year *t*–2, when consumers are still healthy, we hypothesize an overpayment. In year *t*−1 (when signals of the chronic disease start to occur) and years *t*+1 and *t*+2 (when the chronic disease is present, though stable), we hypothesize an underpayment. The actual patterns in spending and RA payments remain an empirical question for the quantitative analyses in this study.

The key difference between Scenarios B and C is the moment consumers take into account their change in health status in choosing a health insurance plan. In Scenario B, consumers respond to the change in health status *after* year *t* (at the start of year *t*+1). This scenario could be expected for diseases that arise acutely (in year *t*), like heart failure or a cancer diagnosis. These are difficult to predict and cannot be anticipated when considering an insurance plan for the upcoming year. In this case, the expected underpayment for insurers that can be avoided through the disruption of the choice of insurance product by consumers is the payment gap shown in years *t*+1 and *t*+2 (and potentially the years after).

In Scenario C, consumers anticipate their imminent change in health status when choosing an insurance plan for year *t*, so before diagnosis. This scenario is more likely for predictable chronic diseases (e.g., due to accumulating health problems and examinations) where consumers might anticipate the onset of the chronic disease when selecting their new insurance plan, or when the eventual diagnosis is made late (the first reason why prospective RA may fall short to compensate these individuals, as explained in the Introduction). The avoidable underpayment for insurers in this scenario, shown by the payment gap in year *t*, is notably larger than in year *t*+1. Moreover, if the individual does not leave the insurer at the next opportunity, the insurer is faced with an additional year of a potentially avoidable, unprofitable contract. Therefore, the incentives for insurers to distort enrollment of the individuals newly diagnosed with a chronic disease, aggravated by the consumer response, are larger in Scenario C than in Scenario B.

The relevance of looking at RA outcomes and switching behavior of individuals who are newly diagnosed with a chronic disease can be illustrated by the following thought experiment. Imagine a scenario in which the average under/overcompensation for a group of individuals with a specific disease is zero. In that scenario, there are no selection incentives regarding this group as a whole. However, there can still be selection incentives regarding subsets of this group, for example, when newly diagnosed individuals are unprofitable to insurers and take into account their health change when switching insurance plans. In that scenario, despite the average fair compensation for spending of the disease group, insurers will prefer to deter the enrollment of newly diagnosed individuals.

This section demonstrates the relevance of two empirical questions in relation to the incentives for insurers to select against individuals with new chronic diseases: (1) to what extent do mean RA payments align with mean spending for this group and (2) if and when do these individuals respond to their change in health status when choosing an insurance plan?

## Context, Data, and Methods

The approach taken for the analyses of this study comprises three steps: (1) identifying individuals who undergo a health shock in year *t* (group g), (2) tracking patterns in mean spending and mean RA payments for group g in the period of year *t*−2 to *t*+2, and (3) tracking whether and when individuals of group g respond to the change in health status in the period of year *t*−2 to *t*+2 through insurer switching as an indicator. This section describes the context of the RA model used in our analyses, as well as the data and methods used.

### Context and Data

The data for this study come from the Dutch basic insurance system, which is based on regulated competition among private insurers, with a mandatory insurance uptake for consumers. Regulatory aspects include an open enrollment policy for every new calendar year, a standardized benefits package, and premium rate restrictions. Aspects of competition include a free consumer choice of insurer and plan type (in terms of deductible level, provider network, and coverage of out-of-network spending) and instruments for insurers to enhance efficiency. The latter includes selective contracting of health care providers and freedom with respect to provider-payment methods. In 2017, the central year of our analysis, 15 insurers were active on the Dutch market, offering a wide selection of insurance plans (58 in total). While most insurers offered a variety of plans with different provider networks, out-of-network spending coverage, options for voluntary deductibles, and supplementary insurance plans, others exclusively specialized in cheaper plans. As a result, for individuals enrolled in a less generous plan with an insurer that offers a variety of plans, switching within the insurer is a viable option. Those enrolled in a less generous plan from a single-plan insurer will have to switch insurers if broader coverage is desired, for instance, when becoming chronically ill.

Moreover, a prospective RA system^4^ is in place to compensate insurers for predictable variation in health spending, thus mitigating incentives for risk selection. Recent studies have shown that the Dutch RA model largely compensates for predictable profits and losses, but not completely (Oskam et al., 2023; Withagen-Koster et al., 2023).

For the empirical analysis, two types of data are used: (1) administrative data used for RA and (2) diagnoses from electronic patient records. The first includes information on all individuals enrolled in a basic health insurance plan for the years 2015 until 2019 (*N* = 16.9 m–17.4 m). This administrative information was used for estimating the Dutch RA models for the years 2018 until 2022. Over these years, the model has undergone modifications that increased the total number of risk classes (dummy variables within the risk adjusters) from 193 to 226 (see Table A1 in the Appendix and Van Kleef et al. (2018)).

In addition to the risk adjusters, the data include information on health spending. In the analysis, we follow the standard procedure of annualizing spending and weighing the outcomes with the fraction of the year the individual was enrolled in health insurance.^5^ For the five consecutive years, the data show an upward trend both in terms of spending and in terms of the number of insured individuals. Mean spending in 2015 stood at €2,285, rising to €2,487 in 2019 (an 8.8% increase). However, for a fair comparison of the evolution of health spending and compensation over the included years, a correction factor is applied to spending in all years to account for inflation and other external effects. In this case, the year 2017—with a mean spending of €2,355—is taken as the benchmark. For the surrounding years, health spending has been adjusted by a factor equal to the relative difference in mean spending between that year and 2017.

The second type of data used for this study is diagnostic information from electronic patient records from GP practices, acquired from the Nivel Primary Care Database (Nivel-PCD) (Nivel, 2019; Vanhommerig et al., 2025). Specifically, this dataset includes 683 dummy variables (representing 683 diagnoses) that indicate the presence (1) or absence (0) of a health problem in a patient. Health problems are coded according to the International Classification of Primary Care, World Organization of National Colleges, Academies (WONCA) & Academic Associations of General Practitioners/Family Physicians, 2005. Out of the 683 diagnoses, 109 have been labeled as chronic diseases (Nielen et al., 2019). With 1.5 million individuals, these data cover about 10% of the total Dutch population and are considered representative of the overall population for the corresponding years (2015–2019) (Van Kleef et al., 2020). In addition, we generated a rebalancing weight for every individual in the Nivel-PCD using the raking method (Battaglia et al., 2009). The use of this weight guarantees that the prevalences and spending in the sample match those in the total population. In other words, it can be expected that, if the Nivel-PCD had covered the entire Dutch population, the findings would have been similar to those found in the weighted sample.

The data facilitate the identification of individuals who were diagnosed with a chronic disease within the period and the year of first registration of the diagnosis. We clustered specific diseases into overarching groups to indicate broader patient groups for our empirical assessment. Based on the available information, six subgroups are constructed: individuals who, in 2017, were diagnosed with: (1) any chronic disease, (2) a chronic mobility disease, (3) diabetes, (4) a chronic heart disease, (5) a chronic lung disease, or (6) cancer.^6^ Table A2 in the Appendix presents a complete overview of the respective clusters.

Merging of the administrative data with the Nivel-PCD through unique individual-level identification codes enables the tracking of patterns in mean spending, mean RA payments, and insurer switching within the period *t*−2 to *t*+2 for individuals registered with a (new) chronic disease in year *t*.

### Methods

The empirical analysis of this study comprised three steps. In the first step we identified individuals who are diagnosed with a new chronic disease in year *t* using the GP patient records within the Nivel-PCD. Through the multi-year combination of the data, the year in which a diagnosis is first registered was found. Moreover, a distinction was made between individuals who are diagnosed with a new disease in year *t* and were ill before, and those who had no chronic diagnosis before that point, effectively transitioning from healthy to chronically ill. Individuals were assigned to the group that was diagnosed with a (specific) chronic disease in year *t* if that disease was registered in year *t* (2017) and not in year *t*−1 (2016). This distinction separates the six initial subgroups into 12.

For the second step of the analysis, we tracked patterns in health spending and RA payments for the selected subgroups. The Dutch RA models of 2018 through 2022 were replicated using the relevant administrative information (for 2015 through 2019). The resulting individual-level RA compensations were then, on an aggregate level, compared to the mean health spending for the subgroups derived in step 1. The comparison demonstrates the mean financial profits or losses for insurers for the groups for the consecutive years.

Next, as a third step of this research, we tracked if and when consumers switched insurer in the period of year *t*−2 to *t*+2. For the 5 contract years in the data (2015–2019), four measurable moments arise for individuals to adjust or switch their health insurance plan (e.g., before January 2016 for the contract period of 2016). As an indicator for the actual consumer response, we determine the percentage of individuals who switch insurers within the groups of interest, for each switching moment. If this percentage peaks in a specific year, we consider that peak as an indication that people within the group respond to changes in health status through insurer switching. Ideally, we would have performed this analysis at the level of insurance plans and deductible level. Unfortunately, our data contains no information on plan membership and deductible level. Instead, we performed this analysis at the level of insurance companies. This is not a perfect representation of the complete consumer response; the consequences of this limitation are discussed in the “Discussion” section.

In addition to detecting peaks in insurer switching within the 5-year period, it is interesting to quantify the propensity of individuals within a group to switch insurer relative to the rest of the population. If the propensity to switch for a group is relatively high, the incentives for insurers to distort the choice of consumers in that group might be stronger, ceteris paribus (all other things being equal). However, since the onset of a chronic disease can be correlated with other consumer characteristics that may influence switching behavior (e.g., age), an additional analysis is required to determine the true influence of the onset of the chronic disease. Therefore, we applied a stepwise regression model to identify what characteristics (from the administrative data) are statistically significant predictors of switching between health insurers. Since age, sex, and the level of education have been proven to be influential factors in the propensity to switch insurers (see, for instance, van Vliet, 2006 and Boonen et al., 2016), these variables were included. Additional information from the data is used (e.g., socioeconomic status) to find an association with insurer switching, while health-specific information is excluded.

The results from the stepwise regression indicate that age, sex and level of education are indeed relevant factors. Females, younger, and higher educated individuals have a higher propensity to switch insurer than their peers. Moreover, the type of employment, being a student, socioeconomic status, and not living in a long-term care facility are significant predictors of switching between insurers. Table A3 in the Appendix provides information on the specific coefficients of these variables.

After identifying the controlling variables, a logit model was estimated to quantify the propensity to switch for the groups that were diagnosed with a (specific) chronic disease in year *t*. For the four separate windows of opportunity to switch in moment y ( y = before January *t*−1, *t*, *t*+1, or *t*+2), the control variables are supplemented with a yes/no indicator for the respective disease/cluster diagnosed by the GP in year *t*. This process is repeated for all six clusters defined in step 1, for all four moments, illustrating the effect of a given diagnosis on the probability $\pi_{i,y}$ of an individual i to switch insurer at moment y :

$$\pi_{i,y}=\frac{exp(\beta_{1}A_{i,y}+\beta_{2}S_{i,y}+\beta_{3}U_{i,y}+\beta_{4}C_{i}+\ldots )}{1+exp(\beta_{1}A_{i,y}+\beta_{2}S_{i,y}+\beta_{3}U_{i,y}+\beta_{4}C_{i}+\ldots )}$$

where $A_{i,y}$ , $S_{i,y}$ , and $U_{i,y}$ represent the age, sex, and level of education, respectively, for individual i in moment y , and $C_{i}$ indicates whether that individual was diagnosed with a (specific) chronic disease in year *t*. Crucially, $C_{i}$ , holds four layers (I, II, III, and IV): individuals who are diagnosed with the disease in year *t* and were healthy before (group I), individuals who received diagnosis in year *t* and already had a chronic disease (group II), individuals who already had the disease in year *t*−1 and still do in year *t* (group III), and the rest of the population (group IV) as a reference group. The equation is completed by the other statistically significant predictors derived from the stepwise regression (the type of employment, being a student, socioeconomic status, and living in a long-term care facility), which are, for visual simplicity, omitted from the equation.

In summary, the analysis on switching behavior is split in two main steps: (1) checking for peaks in switching percentages *within* the considered groups to indicate (the moment of) a response to the change in health status and (2) running the logit model to quantify whether the individuals of a group switch more frequently than other groups (based on timeliness of diagnosis and versus the general population).

## Results

This section presents the results of the empirical analysis. First, the subdivision of the population into groups of individuals who are newly diagnosed with a chronic disease in year *t* is discussed. After, the trends of health spending, RA payments, and switching behavior for these groups are presented for the period *t*−2 to *t*+2.

### Identifying Individuals Who Undergo a Health Shock

Merging the results of the simulated RA models for the years *t*−2 to *t*+2 (2015–2019) with the respective data from the Nivel-PCD leads to a match for 1,548,441 individuals. To qualify, individuals need to be present, at a minimum, in the data for the years 2016 and 2017, recognizing the diagnosis of a new chronic disease in 2017. Table 1 shows the prevalence of the subgroups in the merged dataset. Based on the dataset, nearly 58% of insured individuals had at least one chronic disease in 2017, according to the GPs included in the Nivel-PCD. Here, common chronic diseases that are relatively manageable in terms of health impact make a significant contribution. For instance, 14.2% of the sample is diagnosed with hypertension without complications, 10.7% is diagnosed with dermatitis / atopic eczema, and 9.6% has asthma (not mutually exclusive).

**Table 1:** Prevalence of the Subgroups Based on Chronic Diseases Identified in Nivel Primary Care Database.

	Number of individuals (%)		
Total	1,548,441 (100%)		
At least one chronic disease	894,354 (58.8%)		
	Total	Previously healthy before diagnosis	Had a chronic disease before diagnosis
Diagnosed with any new chronic disease in year *t*	126,746 (8.2%)	39,013 (2.5%)	87,733 (5.7%)
Diagnosed with any chronic mobility disease in year *t*	15,897 (1.0%)	2,692 (0.17%)	13,205 (0.85%)
Diagnosed with diabetes in year *t*	4,403 (0.28%)	979 (0.063%)	3,424 (0.22%)
Diagnosed with heart disease in year *t*	16,199 (1.0%)	2,181 (0.14%)	14,018 (0.91%)
Diagnosed with lung disease in year *t*	3,427 (0.22%)	590 (0.038%)	2,837 (0.18%)
Diagnosed with cancer in year *t*	8,117 (0.52%)	1,579 (0.10%)	6,538 (0.42%)

Moreover, 126,746 individuals (8.2% of the sample) were diagnosed with a new chronic disease in 2017, of which 39,013 (31%) were not diagnosed with any disease in 2016. Further observations include that 0.52% of the population were diagnosed with any cancer in 2017 (excluding skin cancer) and that only 13% (2,181 out of 16,199) of those diagnosed with a heart disease in 2017 were not diagnosed with any disease before.

### The Evolution of Risk Adjustment Payments and Switching Around the Start of a Chronic Disease

**Figure 2.**
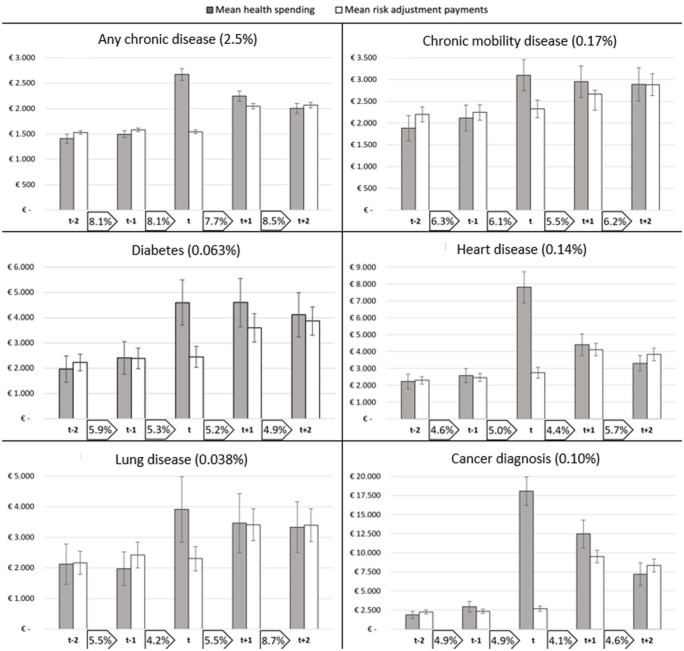
Patterns in Spending, Risk Adjustment Payments, and Insurer Switching for Six Subgroups of Consumers Who Are Diagnosed With a First Chronic Disease in Year t. Note: The six panels in the figure are not mutually exclusive, as individuals can be diagnosed with multiple diseases. The y-axes of the panels differ to facilitate a comparison over the years per disease. The percentage in brackets in the title of a diagram reflects the prevalence of the subgroup in the sample (as shown in Table 2). The lines superimposed on the bars denote 99% confidence intervals. The percentages between the labels on the horizontal axes represent the size of the group that switched insurer from 1 year to the next.^7^ These percentages are separately shown in Figure A1 in the Appendix.

For the identified subgroups of individuals who undergo a health shock, health spending is paired with RA compensations for the relevant years (2015–2019). In Figure 2, both are shown for six subgroups, exclusively covering the individuals who were healthy before the chronic disease was first registered in year *t* (i.e., groups under “previously healthy before diagnosis” in Table 1). The disparity between the bars in the figure for mean spending and mean RA payments illustrates the mean annual payment gap (or surplus) to insurers for enrollees in the specific groups, like Figure 1. The bars include a 99% confidence interval to indicate the statistical significance of the pattern over the years and the difference between spending and compensation. Last, the percentages shown in the boxes between the years represent the percentage of enrollees within the selected group that switches insurer from one year to the next.

**Table 2.** Propensity to Switch Insurer at Four Points in Time, Corrected for Background Characteristics, in Contrast to the Population.

		From year *t*−2 to *t*−1	From year *t*−1 to *t*	From year *t* to *t*+1	From year *t*+1 to *t*+2
		Odds ratio [99% CI]			
Any chronic disease	**I**	1.12 [1.06, 1.17]**	1.14 [1.08, 1.20]**	1.13 [1.07, 1.19]**	1.09 [1.04, 1.15]**
	**II**	0.96 [0.91, 1.01]	0.97 [0.93, 1.02]	0.98 [0.93, 1.03]	0.96 [0.92, 1.01]
	**III**	0.97 [0.95, 0.99]**	0.97 [0.95, 0.99]**	0.97 [0.95, 0.99]**	0.97 [0.95, 0.99]**
Chronic mobility disease	**I**	1.16 [0.94, 1.43]	1.20 [0.97, 1.48]*	1.16 [0.93, 1.44]	1.14 [0.93, 1.41]
	**II**	0.95 [0.84, 1.07]	0.95 [0.84, 1.08]	1.03 [0.91, 1.17]	1.02 [0.90, 1.15]
	**III**	0.92 [0.88, 0.96]**	0.92 [0.88, 0.97]**	0.97 [0.92, 1.02]	0.95 [0.90, 0.99]**
Diabetes	**I**	0.97 [0.68, 1.38]	0.90 [0.62, 1.30]	0.93 [0.64, 1.36]	0.77 [0.52, 1.13]
	**II**	0.96 [0.77, 1.20]	0.85 [0.67, 1.08]	1.08 [0.86, 1.35]	0.88 [0.70, 1.12]
	**III**	0.83 [0.79, 0.88]**	0.81 [0.76, 0.86]**	0.87 [0.82, 0.92]**	0.83 [0.78, 0.88]**
Heart disease	**I**	0.80 [0.61, 1.04]*	0.91 [0.70, 1.17]	0.84 [0.64, 1.10]	0.97 [0.76, 1.24]
	**II**	0.89 [0.78, 1.01]*	0.81 [0.71, 0.93]**	0.82 [0.71, 0.95]**	0.77 [0.67, 0.89]**
	**III**	0.90 [0.85, 0.95]**	0.89 [0.84, 0.95]**	0.86 [0.81, 0.92]**	0.89 [0.84, 0.94]**
Lung disease	**I**	0.94 [0.59, 1.51]	0.74 [0.43, 1.25]	1.02 [0.64, 1.64]	1.46 [0.99, 2.15]*
	**II**	0.79 [0.60, 1.04]*	0.99 [0.77, 1.28]	0.94 [0.72, 1.25]	0.92 [0.70, 1.20]
	**III**	0.83 [0.77, 0.90]**	0.86 [0.79, 0.93]**	0.84 [0.77, 0.92]**	0.84 [0.78, 0.91]**
Cancer	**I**	0.83 [0.61, 1.12]	0.88 [0.65, 1.19]	0.78 [0.56, 1.10]	0.74 [0.53, 1.04]*
	**II**	0.96 [0.80, 1.14]	0.87 [0.71, 1.05]*	0.74 [0.59, 0.93]**	0.75 [0.59, 0.94]**
	**III**	0.85 [0.79, 0.91]**	0.81 [0.75, 0.87]**	0.86 [0.80, 0.93]**	0.90 [0.84, 0.96]**

Note: Group I: Individuals who are diagnosed with the disease in year *t* and were healthy before. Group II: individuals who received diagnosis in year *t* and already had a chronic disease. Group III: individuals who already had the disease in year *t*−1 and still do in year *t*. The reference group in the model is composed of individuals who are excluded from the three respective groups (I, II, and III) per disease. The odds ratios shown result from a logit model that corrects for background characteristics, including age, sex, type of employment, and others. For the full overview, see Table A3 (Appendix). * for *p*<.05; ** for p<.01.

All subgroups start at similar levels of spending, but in the year of diagnosis, the six panels notably diverge. For individuals diagnosed with heart disease or cancer, both relatively acute indications, health spending surges first and then trends down later. For all diseases, prospective RA is, as expected, unable to accurately compensate for the rise in spending in year *t*, but improves in the subsequent years. For all diagnoses, the confidence intervals between health spending and RA payments in year *t* do not overlap, indicating a statistically significant payment gap to insurers in the year of diagnosis. In the years before and after, the gap between spending and compensations is mostly no longer statistically significant. Moreover, in the year prior to diagnosis, a slight rise in health spending is observed for most subgroups, providing an indication of the latent health change or a belated diagnosis by the GP.

In terms of switching behavior from 1 year to the next, some interesting observations can be made. First, the rate of insurer switching is relatively high for the overarching group of individuals who were healthy before and were diagnosed with any chronic disease in year *t* (the top-left diagram).^8^ However, there are no obvious peaks in switching for this group during the period of interest. For the five more specific subgroups, we find switching rates below that of the population (given in footnote 7) and some deviating patterns. Over the five-year period, individuals who were diagnosed with a chronic disease of the heart or lungs see an overall increase in the switching rate, while the opposite holds for the other three subgroups. Still, these numbers provide no convincing signal of a peak in insurer switching in response to the onset of the chronic disease. The exception to this finding is the peak in switching from year *t*+1 to year *t*+2 for individuals who are diagnosed with a lung disease in year *t*.

**Figure 3.**
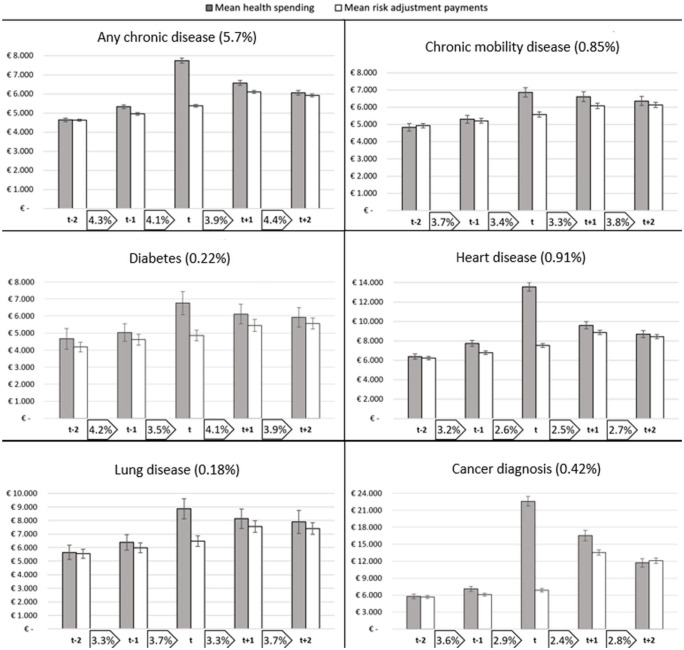
Patterns of Spending, Risk Adjustment Payments, and Insurer Switching for Six Subgroups of Consumers Who Are Diagnosed With a Chronic Disease in Year t on Top of an Existing Chronic Disease. Note: The six panels in the figure are not mutually exclusive, as individuals can be diagnosed with multiple diseases. The *y*-axes of the panels differ to facilitate a comparison over the years per disease. The percentage in brackets in the title of a diagram reflects the prevalence of the subgroup in the sample (as shown in Table 2). The lines superimposed on the bars denote 99% confidence intervals. The percentages between the labels on the horizontal axes represent the size of the group that switched insurer from 1 year to the next. These percentages are separately shown in Figure A2 in the Appendix.

For the other six subgroups of our study, those that were diagnosed with a chronic disease on top of existing chronic unhealth, we find higher starting levels of spending in Figure 3. Nevertheless, we find similar patterns in the evolution of RA payments for these subgroups: statistically significant payment gaps in year *t*. However, these gaps linger in the first year after diagnosis. Only in the case of a cancer diagnosis, a surplus is shown in year *t*+2, albeit without statistical significance. In terms of switching, we find lower levels for all six subgroups than for the population (Footnote 7). For all groups, we find either a decrease in insurer switching or a stable pattern over the years.

The absolute differences in insurer switching between the subgroups and the overall population may be caused by underlying factors. Correcting for these individual-level characteristics such as age and level of education (as discussed in the “Methods” section) provides more insight into the propensity to switch in relation to the diagnosis in year *t*. Table 2 presents the propensity to switch insurers for all subgroups for the four moments to switch, derived from the logit model. For all six clusters, four groups are made (I, II, III, and IV): individuals who are diagnosed with the disease in year *t* and were healthy before (group I), individuals who received diagnosis in year *t* and already had a chronic disease (group II), individuals who already had the disease in year *t*−1 and still do in year *t* (group III), and the rest of the population (group IV) as a reference group.

In Figure 2, higher switching rates were found for the overarching group of individuals who are diagnosed with a first chronic disease, relative to the general population. After correcting for background characteristics, we find that those individuals diagnosed with a chronic disease in year *t* who were healthy before are indeed more likely to switch insurer in all four moments (α = 0.01). Individuals who already were diagnosed with a chronic disease and were diagnosed with an additional disease in year *t* (group II) do not deviate significantly from the population.

However, for our selection of specific (clusters of) diseases we find slight deviations from the earlier patterns when correcting for the background characteristics. Individuals who were diagnosed with a chronic mobility disease and were healthy before are more likely to switch insurer before January of year *t* than the general population, although lower percentages were reported in Figure 2. Healthy individuals diagnosed with heart disease in year *t* have a lower propensity to switch before January *t*−1, and those diagnosed with such a disease in general have a lower likelihood to switch throughout the considered period. For individuals diagnosed with cancer, we find a lower propensity to switch before and after diagnosis. Only for those diagnosed with a chronic lung disease, while being healthy before, we find a higher likelihood to switch insurer *after* diagnosis (before January *t*+2) (OR: 1.46). In general, we find individuals who already were chronically ill to be less likely to switch insurers than the overall population. Except for the group diagnosed with a chronic mobility disease, we find no evidence of an increase in insurer switching behavior in anticipation of, or in response to, the diagnosis of a specific, or clustered, chronic disease. Also, at a 99% confidence level, it cannot be claimed that for any of the subgroups shown here in any of the switching moments, individuals are more likely to change insurer than in the prior year (because of the structural overlap in confidence intervals between the considered moments). In summary, for the (clusters of) diseases we selected, we find no elevated switching rates in the years surrounding diagnosis (except for the chronic mobility disease group before year *t* and the lung disease group before year *t*+2), while we do find a significant effect for the group of individuals who are diagnosed with a first chronic disease in general. This implies that for other diseases not included in our clusters, elevated switching rates should be found. Additional analysis confirms the presence of above-average switching rates for individuals diagnosed with relatively less severe and more common chronic conditions like asthma and eczema.

## Discussion

In this research, we tracked the 5-year evolution of health spending, compensations to insurers through RA, and insurer-switching behavior for individuals who are diagnosed with a new chronic disease. The dynamics between the three provide an indication of the incentives for insurers to distort the natural enrollment of individuals who are diagnosed with a chronic disease. Using multi-year GP data from the Nivel-PCD, we identified subgroups of the Dutch population who were diagnosed with a chronic disease in 2017 and found all subgroups to be substantially underpaid by the prospective RA model in place in the year of diagnosis: a logical consequence of the belated recognition of the onset of a chronic disease in prospective RA. In the subsequent years, RA compensations increase but often do not completely cover health spending.

We argued that the incentives for insurers to engage in risk selection toward individuals who are first diagnosed with a (new) chronic disease depend on the presence of a payment gap, and whether and when these individuals anticipate the health shock in their plan selection. Through examination of the insurer switching behavior of individuals—as an indicator of consumer response—we find no obvious peaks in switching in years before or after the chronic disease was first diagnosed. However, we do find that—on average—people first diagnosed with a chronic disease are more likely to switch insurer than the rest of the population, both before and after the first diagnosis. For our selection of specific diseases, however, we do not find strong indications that newly diagnosed individuals act in anticipation of, or in response to, their change in health status.

Importantly, the findings with respect to consumer response are subject to caveats. As mentioned in the “Methods” section, switching between insurers as our only indicator for responsive behavior is incomplete. In response to an imminent health change, a logical action by individuals could be to adjust their voluntary deductible or switch to a broader insurance plan offered by the same insurer. Therefore, the insurer switching rates used in our analyses are likely underestimations of actual switching behavior. That might be a reason why our findings do not directly match earlier studies on the association between health plan enrollment and health status. Another reason could be that the level of basic coverage in the Netherlands is quite substantial as a baseline, or that consumers may well be enrolled in a relatively generous plan already. Both reduce the need for a change of insurance plan in response to a diagnosis. Despite the standardized basic benefit package in the Netherlands, insurance products may differ on the level of contracted providers. Packages with unrestricted access to providers are typically more expensive. A more thorough analysis of the plan package design and deductible choice of individuals in this context could find results that align more with earlier studies. Moreover, a related element affecting our findings is the fact that the results depend on the Dutch context and the available health plans on the market during the specific years included in the research. Within that context, the design of health plans can be the consequence of actions of selection by both insurers and consumers. Therefore, switching behavior and plan design share an endogenous relationship. This dynamic should be considered in further research, for instance, by incorporating the plan attributes before and after switching.

Another limitation of our analysis is the focus on a subset of chronic diseases. An interesting direction for future research is to track spending, RA payments and switching for other diseases. If specific diseases are in fact problematic in the light of selection incentives (e.g., a payment gap and an active anticipation of the health change by consumers), regulators could consider concurrent RA for that disease (e.g., diagnoses from year *t* are used to “predict” health spending in year *t*) to reduce the potential incentives for risk selection by insurers. However, concurrent RA is not without flaws either: the endogenous link between health care consumption and financial compensation in the same year may reduce incentives for insurers to control costs (Van de Ven & Ellis, 2000). Generally, regulators could think of a hybrid system with prospective risk adjusters for diseases that are unlikely to be anticipated by consumers and concurrent risk adjusters for diseases that can be anticipated to reduce incentives for risk selection.

In sum, by tracking spending and RA payments over the period *t*−2 to *t*+2 for individuals diagnosed with a new chronic disease in year *t*, we find a substantial payment gap in year *t* and, to a lesser extent, in prior and/or subsequent years. Our analysis of insurer switching shows that—on average—people first diagnosed with a chronic disease are more likely to switch insurers than others. This could indicate that consumers respond to their change in health status, which could aggravate the selection incentives for insurers that result from the observed payment gaps. Circling back to the central question of this study: for diseases that are statistically significant undercompensated by the RA model in year *t*+1 and/or *t*+2, we argue that the model does not compensate adequately enough. Competing health insurers can potentially steer the enrollment behavior of individuals diagnosed with such unprofitable diseases when that information becomes known to insurers. If these individuals are concentrated at specific insurers, due to enrollment in specific health plans, no level playing field for insurers may exist. To assess the extent to which undercompensations in year *t* are truly problematic, a deeper analysis of switching and enrollment behavior is required, in addition to studying selection strategies by insurers. Further research is therefore needed to analyze switching patterns at the level of plan attributes (e.g., deductible level and provider network) and repeat our analysis for (other) diseases of interest.

## Figures and Tables

**Figure A1.**
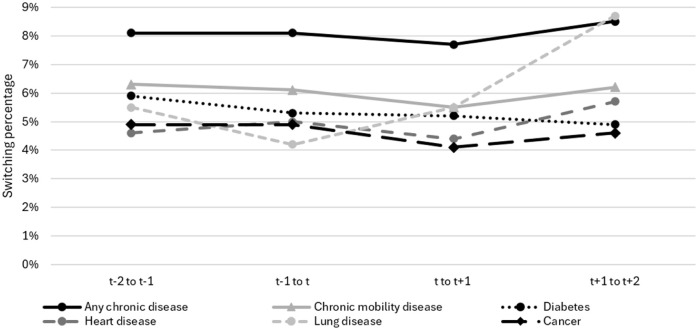
Switching Percentage Over Four Windows for Individuals Who Are Diagnosed With a Chronic Disease in Year t and Who Were Healthy Before.

**Figure A2.**
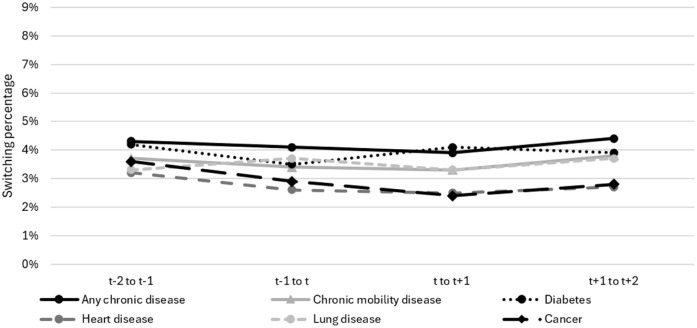
Switching Percentage Over Four Windows for Individuals Who Are Diagnosed With a Chronic Disease in Year t and Who Were Already Diagnosed With a Chronic Disease Before.

## Appendix

**Table A1.** Risk Adjusters in the Dutch Risk Adjustment Models of the Years 2018 Until 2022 and Information on Spending for the Respective Years Underlying the Models.

	Number of risk classes (dummy variables)				
	*2018*	*2019*	*2020*	*2021*	*2022*
**Risk adjusters**					
Age interacted with sex	42	42	42	42	42
Pharmacy-based cost groups	34	38	38	39	43
Diagnosis-based cost groups				27	27
Primary diagnosis-based cost groups	16	16	16		
Secondary Diagnosis-based Cost Groups	8	8	8		
Durable medical equipment cost groups	11	11	11	15	15
Source of income/education interacted with age	25	25	36	36	36
Zip-code clusters for region	10	10	10	10	10
Socioeconomic status	12	12	12	12	12
Household size interacted with age	13	13	13	13	13
Multiple-year high-cost groups	9	9	9	9	9
Physiotherapy-diagnosis cost groups	5	5	5	5	5
Home care spending in the previous year	8	10	10	10	10
Multiple-year pharmaceutical costs *and* historical somatic morbidity					4
**Total**	**193**	**199**	**210**	**218**	**226**
**Information on spending and age**	*2015*	*2016*	*2017*	*2018*	*2019*
Mean spending in the population	€2,285	€2,352	€2,355	€2,408	€2,487
Mean spending after correction	€2,355	€2,355	€2,355	€2,355	€2,355
Number of insured individuals	16,945,802	17,036,618	17,128,725	17,256,295	17,365,571
Number of insured years	16,672,147	16,749,830	16,839,948	16,951,700	17,041,789
Under 18	20.4%	20.2%	19.9%	19.7%	19.5%
18–34 years of age	20.4%	20.4%	20.6%	20.9%	21.0%
35–54 years of age	27.9%	27.5%	27.1%	26.7%	26.3%
55–69 years of age	19.3%	19.4%	19.4%	19.4%	19.5%
70–79 years of age	7.7%	8.0%	8.4%	8.8%	9.1%
Aged 80 or above	4.4%	4.5%	4.6%	4.6%	4.8%

**Table A2.** Overview of the Clustered Diseases for the Defined Subgroups.

Cluster	Diseases included
Mobility	(Seropositive) rheumatoid arthritis (L88), Osteoarthrosis of hip (L89), Osteoarthrosis of knee (L90), Osteoarthrosis otherwise (L91), Osteoporosis (L95).
Lung	Chronic bronchitis/bronchiectasis (R91), Chronic obstructive pulmonary disease (R95).
Heart	Ischemic heart disease with angina (K74), Acute myocardial infarction (K76), Heart failure (K77), Pulmonary heart disease (K82), Hypertension complicated (K87), Stroke / cerebrovascular accident (K90), Atherosclerosis (K91), Cardiovascular disease otherwise (K92).
Cancer	Malignancy, not otherwise specified (A79), Hodgkin’s disease / lymphoma (B72), Leukemia (B73), Malignant neoplasm blood otherwise (B74), Malignant neoplasm stomach (D74), Malignant neoplasm colon/rectum (D75), Malignant neoplasm pancreas (D76), Malignant neoplasm digestive otherwise / not otherwise specified (D77), Malignant neoplasm nervous system (N74), Malignant neoplasm bronchus/lung (R84), Malignant neoplasm respiratory otherwise (R85), Malignant neoplasm thyroid (T71), Malignant neoplasm kidney (U75), Malignant neoplasm bladder (U76), Malignant neoplasm urinary otherwise (U77), Malignant neoplasm cervix (X75), Malignant neoplasm breast female (X76), Malignant neoplasm female genital otherwise (X77), Malignant neoplasm prostate (Y77), Malignant neoplasm male genital otherwise (Y78).

**Table A3.** Odds Ratios for the Background Characteristics on Insurer Switching for the Four Switching Moments Considered, Resulting From the Stepwise Regression.

		From year *t*–2 to *t*–1	From year *t*–1 to *t*	From year *t* to *t*+1	From year *t*+1 to *t*+2
Variable	Description	Odds ratio			
Sex	*Man*	0.945	0.93	0.913	0.907
	*Woman*	1	1	1	1
Age	*Under 18*	4.968	5.16	5.815	6.745
	*From 18 to 34*	6.726	7.129	7.513	8.137
	*From 35 to 54*	4.199	4.481	4.764	5.558
	*From 55 to 69*	2.877	2.966	3.083	3.672
	*From 70 to 79*	1.506	1.61	1.668	1.844
	*80 or above*	1	1	1	1
Source of income	*Unable to work, unemployed or government allowance.*	0.828	0.874	0.928	0.856
	*Student*	1.091	1.089	1.094	1.056
	*Self-employed*	1.132	1.108	1.168	1.162
	*High educated*	1.552	1.468	1.631	1.646
	*Reference group*	1	1	1	1
Socioeconomic status	*Very low*	1.036	1.183	1.125	1.028
	*Low*	1.035	1.144	1.078	1.037
	*Mid*	1.051	1.105	1.093	1.085
	*High*	1	1	1	1
Living in long-term care facility	*Yes*	0.545	0.595	0.497	0.519
	*No*	1	1	1	1

## Notes

1. We use insurer switching as a proxy for the consumer response. This period includes four annual switching windows. In the “Methods” section we provide more details on the operationalization of this proxy, and we elaborate on the consequences of its use in the Discussion.

2. For more examples on risk adjustment evaluation studies, see: Bergquist et al. (2019); McGuire, Zink, and Rose (2021); Xu et al. (2021).

3. For example, systems with multiple, largely different insurance coverage options (e.g., in the United States) will have notable inter-system switching in addition to plan switching within the system.

4. The risk adjustment system includes separate models for somatic care, mental care, and out-of-pocket spending under the mandatory deductible. In this paper, we focus on the former, which covers roughly 90% of total spending under the basic health insurance.

5. Individuals may, for specific reasons, have canceled their health insurance at any point during the year (or have enrolled after January 1). Therefore, individual-level spending is annualized and weighted by the fraction of the year an individual was enrolled. For example, spending accumulated by individuals who were enrolled for half of the year is doubled and attributed a weight of 0.5 in the analyses.

6. Subgroups 3 through 6 are comprised of clustered diagnoses. Ambiguous diagnoses were excluded from the cluster to protect its validity.

7. The overall switching rates in the population are 6.8%, 7.0%, 6.4%, and 7.3% for the respective moments.

8. The overarching group contains all individuals who were diagnosed with any of the 109 chronic diseases in 2017 and were healthy in 2016. The switching rates of this group exceed those of the general population (Footnote 7) and the other subgroups (Figure 2). Additional research points out that the elevated switching rates come from relatively frequent, less severe chronic diseases such as asthma and eczema, which are more prevalent among younger individuals who tend to switch more frequently. The proven correlation between age and switching behavior helps to explain the result in Figure 2.
